# A hybrid tendon-driven continuum robot that avoids torsion under external load

**DOI:** 10.3389/frobt.2025.1576209

**Published:** 2025-05-14

**Authors:** Maria Paula Huertas Niño, Mohamed Boutayeb, Dominique Martinez

**Affiliations:** ^1^ Aix Marseille University, CNRS, ISM, Marseille, France; ^2^ UMR7039 Centre de recherche en automatique de Nancy (CRAN), Vandoeuvre LesNancy, Lorraine, France

**Keywords:** tendon-driven continuum robot, revolute joints, torsional deformation, constant curvature model, agricultural robotics, harvesting

## Abstract

Tendon-driven continuum robots usually consists of several actuators and cables pulling a flexible backbone. The tendon path alongside the backbone allows to perform complex movements with high dexterity. Yet, the integration of multiple tendons adds complexity and the lack of rigidity makes continuum robots susceptible to torsion whenever an external force or load is applied. This paper proposes a reduced complexity, hybrid tendon-driven continuum robot (HTDCR) that avoids undesired torsion under external load. Bending of the HTDCR is achieved from a single tendon with lateral joints alongside the backbone acting as mechanical constraint on the bending plane. A rotary base then provides an additional degree of freedom by allowing full rotation of the arm. We developed a robot prototype with control law based on a constant curvature model and validated it experimentally with various loads on the tip. Body deviation outside the bending plane is negligible (mm range), thereby demonstrating no torsional deformation. Tip deflection within the bending plane is smaller than the one obtained with a 4-tendon driven continuum robot. Moreover, tip deflection can be accurately estimated from the load and motor input which paves the way to possible compensation. All together, the experiments demonstrate the efficiency of the HTDCR with 450 g payload which makes it suitable in agricultural tasks such as fruit and vegetable harvesting.

## 1 Introduction

Tendon-driven continuum robots (TDCRs) are characterized by their continuous, flexible structure actuated by tendons or cables that run along the body, enabling smooth and multi-directional movements that depend on factors such as the number of cables, cable routing, number of sections, among others. There has been a growing interest in such highly adaptive mechanical systems capable of operating in unpredictable or constrained spaces with soft interaction with the enviroment, such as encountered in medical or agricultural applications (Russo et al., 2023; Bai et al., 2024). Medicine is currently a key focus of interest and research for TDCRs as their continuum structure can adapt to the human body made up of soft and deformable organs. Additionally, they can be miniaturized to millimeter-scale diameters and centimeter-scale lengths, providing a significant advantage over conventional robots with rigid links in minimally invasive procedures (Veiga et al., 2020). TDCRs have been deployed as steerable cardiac catheter with 3.8 mm diameter and a NiTi rod backbone (Camarillo et al., 2008), for cardiovascular intervensions composed of rolling contact joints with 4.65 mm diameter (Kim et al., 2018), in orthopedic surgery (Alambeigi et al., 2019), intracerebral hemorrhage evacuation (Yan et al., 2022), among others.

Agriculture is an emerging field for TDCRs, holding great potential for navigating through crop fields without causing damage to the plants (Armanini et al., 2024). However, TDCRs flexibility, also implies lower stiffness materials, which can pose challenges in achieving precise positioning under load or in responding to external forces without deformations. This limitation can be particularly problematic for harvesting where, despite the relatively low weight of common fruits (on average, 150 g for peaches, 120 g for pears, and 200 g for apples), external forces can still induce undesired bending or twisting that disrupts the robot’s accuracy. As a result, achieving consistent and controlled interaction with the environment may require compensatory mechanisms, such as advanced feedback systems or structural reinforcements, to mitigate these unintended movements and improve handling precision.

There have been efforts to counteract or compensate the effects of external loads on TDCRs, such as deflection, torsional deformation, buckling and slack cable, to enable their use at larger scales making them more suitable for applications in fields such as agriculture. In (Yeshmukhametov et al., 2020; Yuan and Li, 2018), a pre-tensioning mechanism was used to avoid cable slack in a TDCR with universal joints that aims to harvest cherry tomatoes. There have also been proposals to incorporate mechanisms that restrict the robot’s movements, aiming to enhance stability and control under external loads such as a sliding mechamism as in (Tokunaga et al., 2017), also adding an extra cable attached directly to the tip, without routing it through the tendon paths as a means to provide a compensation (Childs and Rucker, 2021). Efforts have also been made to implement different materials and joints that increase resistance, as demonstrated in (Xiao et al., 2024), where a TDCR with two segments (each 80 mm in length and 70 mm in diameter) utilizes a shape memory alloy (SMA) spring and a gooseneck structure achieving a resistance capacity of 5 N.

Similarly, a novel joint design have been proposed as in (Dong et al., 2015) and (Yang et al., 2021), where the implementation of twin-pivot structures using NiTi rods as elastic joints, enhanced resistance under external loading has been observed. However, such mechanisms have been typically fabricated with a small diameter/length ratio, 15/280 (mm) and 17/150 (mm) respectively, limiting their application to small-scale robotic systems or structures that do not require extensive reach or high load capacity. In Dong et al. (2017), Wang et al. (2021), Yang et al. (2024), tests were conducted on multi-segment TDCRs for inspection/repair of engines by implementing twin-pivot structure and contact-aided compliant mechanisms design concepts with diameter/length ratio of 13–40/1,270 (mm) and 14–16/384 (mm) respectively with load capacity of up to 200g and 300 g with body deformations, showing that implementing longer structures with wider diameters does not necessarily lead to an increase in load capacity.

This paper focuses on a TDCR that could be used in agricultural tasks like fruit or vegetable harvesting, pest or disease detection, pesticide or fertilizer spray, among others. In these tasks, size and precision requirements for the robot are less critical than in medical applications. Most important is that the TDCR should have the ability to carry loads with some flexibility to navigate through crop fields. To prevent torsional deformation under load while maintaining bending flexibility, we propose a hybrid tendon-driven continuum robot (HTDCR) with a rotary base and rigid links alongside a flexible backbone. A unique tendon path allows bending in a plane whose direction is given by the rotary base. Because rigid-link structures offer relatively high precision and load capacity as compared to compliant joints, the HTDCR does not experience any torsion or deviation outside the bending plane.

## 2 Materials and methods

### 2.1 HTDCR principle and mechanical design

In tendon-driven continuum robotics, typically flexible materials are used as joints and/or the backbone giving them the capacity of enabling complex movements actuated by cables. Depending on factors such as number of cables and cable routing, the robot can bend in one or several directions. Standard TDCRs require at least two tendons for planar bending and three tendons for spatial bending (Rao et al., 2021). TDCRS are, however, susceptible to deformation under external loads or forces affecting their precision. In classical robotics, revolute joints are commonly used where controlled and precise rotational movements are required. Revolute joints allow relative rotation between two parts joined around a fixed axis, restricting motion to a single degree of freedom, meaning that the body can rotate but cannot translate in other directions.

**FIGURE 1:**
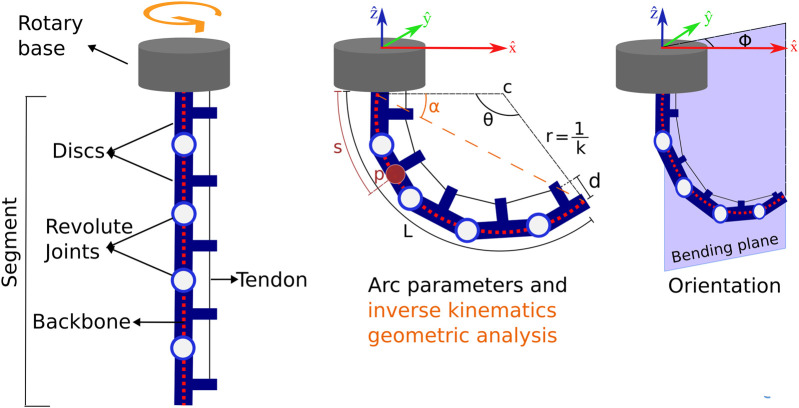
Schematics of the proposed hybrid cable-driven continuum robot (HTDCR) implementing revolute joints and a rotary base. In the constant curvature model, the shape of the backbone is defined by the direction of bending (Φ) , the bending angle (θ) and curvature constant (κ) . The fixed parameters are the length L of the backbone and the distance d of the tendons with respect to the backbone.

By combining these two concepts, a hybrid tendon-driven continuum robot (HTDCR) is proposed with revolute joints placed laterally along a flexible backbone. A unique tendon path allows bending the backbone in a plane whose direction is given by the rotary base (Figure 1).

**FIGURE 2:**
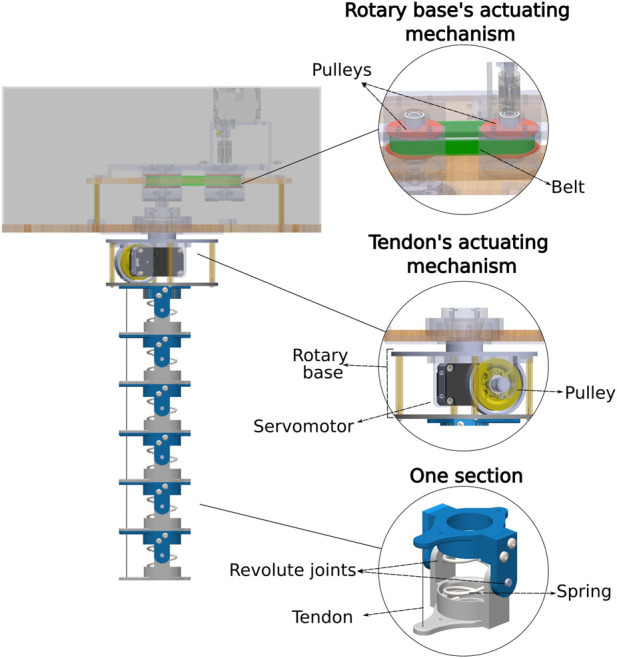
Mechanical design of the proposed HTDCR which implements a belt and pulley system for rotation and a pulley and motor system to actuate the tendon.

A prototype of the HTDCR was built from a backbone with 6 sections (Figure 2), each one having a steel spring (inner diameter of 25.6 mm, length of 42 mm, wire diameter of 1.8 mm and spring constant of 2.119 N/mm) encapsulated between two 3D printed discs with ToughPLA. The springs provide stiffness to the robot body (Xing et al., 2021; Santiago et al., 2015; Yoon and Yi, 2009; Gao et al., 2015; Visentin et al., 2019; Qi et al., 2024) and its hollow center allows the integration of cables, tubes, and sensors within their internal structure. Aluminium pins are placed laterally and maintained with snap rings so that the backbone can resist to torsional stress, ensuring more stability during operation when an external force or load is added on the tip. Additionally, the revolute joints create a mechanical constraint that maintains the length of the springs at any time. Thus, there is no elongation or compression of the springs when the tendon is actuated, which results in bending motion only with a stiffness at each joint.

To enable bending in one direction, one tendon path is added to the discs and incorporates a steel cable with a diameter of 0.8 mm parallel to the backbone. The tendon is fixed to the tip and wraps around a pulley attached to a DYNAMIXEL XL-430-W250-T servomotor. An adjustment is made to the initial position of the motor to ensure that the cable is taut from the start. Then, the cable remains taut in any bending position because of the revolute joints that prevent elongation or compression of the backbone. This tendon servomotor is fixed to a rotating plate, which allows the body to rotate 360°, providing an additional degree of freedom to the robot. To enable rotation, another DYNAMIXEL XL-430-W250-T servomotor is coupled to a pulley (30T and 48 T) and GT2 belt system is mainly used for impact absorption and motion transmission. The current design does not support multiple rotations of the rotary base. This is to prevent twisting of the power and data cables that supply the servo motor in the rotary base. Moreover, a 360° rotation covers the entire workspace and there is no requirement for having multiple turns. Yet, if needed in future implementations, the use of a rotating connector can accommodate continuous rotation without causing entanglement.

### 2.2 Constant-curvature kinematic model

A constant curvature model was implemented for the HTDCR due to its simplicity. It assumes that the body has a constant curvature and neglects gravitational and torsional effects, in addition to the effects that may also be caused by external forces and the weight of the robot. Despite its simplicity, this geometric model has been shown to be effective and is widely used in continuum robotics (Webster and Jones, 2010; Li et al., 2018; Wang et al., 2013). It is worth noting that the model complexity for the HTDCR is even reduced as compared to (Webster and Jones, 2010; Li et al., 2018; Wang et al., 2013) because bending the body is performed with a single tendon and the bending orientation is directly obtained from the rotary base ( ϕ proportional to the base motor position). The kinematics is decomposed into three operating spaces: joint, configuration and task spaces. The joint space is defined by the tendon length $L_{\mathrm{tendon}}$ and the direction of bending ϕ . The configuration space, which describes the shape of the backbone, is defined by arc parameters (i.e., bending angle θ and curvature constant κ , Figure 1). The task space is defined by the position and orientation of any point of the backbone.

The forward kinematics determines the position and orientation of any point of the backbone based on the change in tendon length $\Delta L_{\mathrm{tendon}}$ and the bending plane direction ϕ . The latter is proportional to the motor position of the rotary base with proportional coefficient 1.6 identified from the pulleys’ characteristics. From the current tendon length $L_{\mathrm{tendon}}=L-\Delta L_{\mathrm{tendon}}$ , where L is the fixed length of the backbone, the arc parameters are obtained in Equation 1.

θ=κ L (1)

$$\kappa =\left|\frac{L-L_{\mathrm{tendon}}}{L\;d}\right|$$ (2)

with θ the bending angle and κ the curvature constant (Figure 1). From the arc parameters, an homogeneous transformation matrix can be applied in any point s of the backbone to transition to the task space and determine the system coordinates. The homogeneous transformation matrix writes as follows (Webster and Jones, 2010):

$$T=\left[\begin{array}{cccc} \cos\left(\phi \right)\cos\left(\kappa s\right) & \sin\left(\phi \right) & \cos\left(\phi \right)\sin\left(\kappa s\right) & \frac{\cos\left(\phi \right)\left(1-\cos\left(\kappa s\right)\right)}{\kappa }\\ \sin\left(\phi \right)\cos\left(\kappa s\right) & -\cos\left(\phi \right) & \sin\left(\phi \right)\sin\left(\kappa s\right) & \frac{\sin\left(\phi \right)\left(1-\cos\left(\kappa s\right)\right)}{\kappa }\\ \sin\left(\kappa s\right) & 0 & -\cos\left(\kappa s\right) & -\frac{\sin\left(\kappa s\right)}{\kappa }\\ 0 & 0 & 0 & 1 \end{array}\right]$$ (3)

The entries in T depend on the arc length (s) between the base and any point (p) of the backbone, where s∈(0 L) . The Cartesian coordinates of any point s of the backbone are directly obtained from the last column in T , i.e., $\left(X,Y,Z\right)=\left(T\left(1,4\right),T\left(2,4\right),T\left(3,4\right)\right)$ . However, when the robot is fully extended facing downward, these coordinate calculations do not apply given that κ=0 . Instead, one has (X,Y,Z)=(0,0,−s) .

The inverse kinematics determines the change in tendon length and bending direction for a given tip position $\left(X_{\mathrm{tip}},Y_{\mathrm{tip}},Z_{\mathrm{tip}}\right)$ . A geometrical analysis of the bending plane (Figure 1) leads to Equations 4, 5.

$$\theta =\pi -2\alpha \;\text{with }\alpha =\arctan\left|\frac{Z_{\mathrm{tip}}}{X_{\mathrm{tip}}}\right|$$ (4)

$$\kappa =\frac{1}{r}=\frac{\theta }{L}$$ (5)

The tendon length can then be determined from the above arc parameters and the forward kinematics (Equation 2). Finally, the direction of the bending plane is given by Equation 6

$$\phi =\arctan\left(\frac{Y_{\mathrm{tip}}}{X_{\mathrm{tip}}}\right)$$ (6)

### 2.3 4-Tendon driven continuum robot (4TDCR) for comparison with HTDCR

**FIGURE 3:**
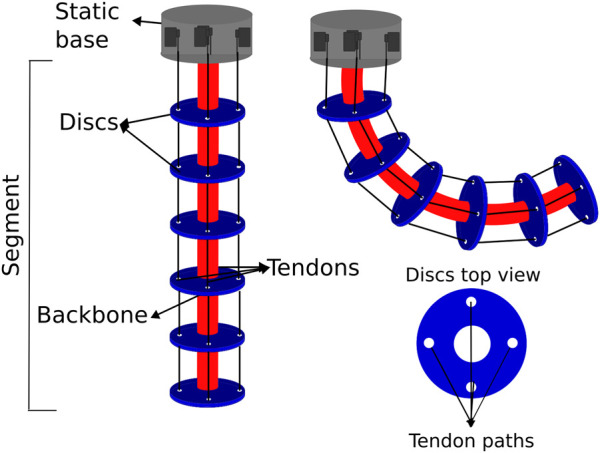
Schematics of the 4TDCR. The backbone is shown in red. as for the HTDCR, it is made of 6 sections, each one having the same steel spring. The sections are separated by discs with 4 holes for the tendon paths.

A standard prototype was built based on the design principles for TDCRs outlined in Section 2.1. The aim of this 4TDCR prototype is to allow comparison with the HTDCR. The 4TDCR consists of a spring backbone connected by spaced discs achieving its actuation through four tendons parallel to the backbone, and attached to the tip of the robot arm (see Figure 3). Each tendon is spaced at 90° intervals and controlled independently by a servomotor, thus allowing bending in any direction. Despite design differences between 4TDCR and HTDCR, both prototypes share the same dimensional parameters (i.e., L and d in Figure 1) and springs so that the stiffness and cable tension are similar, thus maintaining consistency in the structural design.

Additionally, a constant-curvature kinematic model was implemented for controlling the 4TDCR. The 4TDCR model has been developed and validated in previous works (Rao et al., 2021; Webster and Jones, 2010; Li et al., 2018; Neppalli and Jones, 2007). Specifically, the arc parameters depend on the length of each tendon, as the differential actuation of the tendons generates bending. Although related, the constant-curvature model for the HTDCR is simpler as it depends on a single tendon (Section 2.2).

## 3 Results

### 3.1 Validation of the constant curvature model for the HTDCR

**FIGURE 4:**
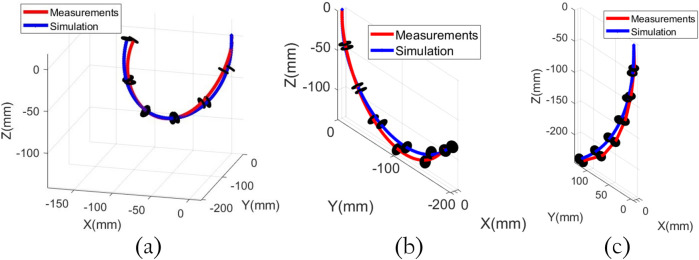
HTDCR measurements vs. simulations of the constant curvature model for three different bendings. **(a)** Maximum bending **(b,c)** intermediate bendings.

To validate the constant curvature model, measurements were obtained on the real HTDCR with a motion capture system (Qualisys 6 Miqus cameras and 14 reflective markers placed along the body). We compared the measurements to simulations of the forward kinematic model (last column of T in Equation 3). Figure 4 shows three examples of spatial bending for the simulations and HTDCR measurements. The error at the tip was computed for 14 different bending positions where each position was repeated 3 times. The errors range from 6mm to 24 mm which is in agreement with errors reported in other studies for open-loop control based on constant-curvature models (Qi et al., 2024; Amanov et al., 2021). Although out of the scope of the paper, it is worth noting that the precision could be improved by using closed-loop control as in (Qi et al., 2024).

### 3.2 HTDCR maximum payload estimation

**FIGURE 5:**
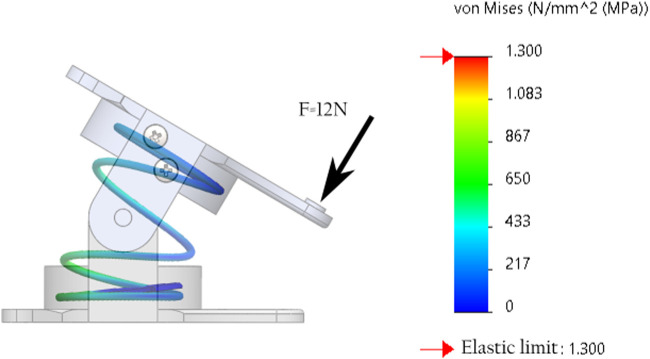
SolidWorks simulation of one section of the arm under maximum required beding force of 12N .

A prerequisite to experiments under load is to characterize the maximum load the HTDCR can support. The force required to induce maximum bending of the body without load is estimated to $F_{\mathrm{body}}=12N$ . This estimation was obtained from SolidWorks simulations of large displacements Finite Element Method (FEM) for one individual section of the arm, focusing on the spring under bending loads. Figure 5 shows the stress of one section of the arm with a 12N actuation force. We can see that the stress supported by the spring ranges from 0MPa to 710MPa which is well below the elastic limit of 1300MPa for the steel material (EN 10270–1) of the spring.

The bending of individual sections was then replicated for the whole arm and validated experimentally by measuring the force for maximum bending of the arm with a dynamometer (KERN HDB 5K5N). Then, maximum force capacity $F_{\max}$ is found by considering a stall torque for the motor of 1.5Nm. However, an operating torque of $\tau_{\mathrm{motor}}=1Nm$ was chosen to ensure a safe margin, with a pulley radius $r_{\mathrm{pulley}}=0.013m$ , and a safety factor SF=1.4 , chosen as a balance between ensuring reliability and avoiding excessive oversizing, while considering potential load variations and efficiency losses. This leads to Equation 7.

$$F_{\max}=\frac{\tau_{\mathrm{motor}}}{SF\;r_{\mathrm{pulley}}}-F_{\mathrm{body}}=43N$$ (7)

This maximum force equals to 4,379 g-force, and, dividing by 9.81 $m/s^{2}$ , ones gets a maximum load capacity of approximately 446 g. This theoretical value was confirmed experimentally by adding weights to the tip in increments of 45 g until the physical maximum capacity of the motors was reached, in which we found a maximum load of 450 g.

### 3.3 Body deviation analysis

**FIGURE 6:**
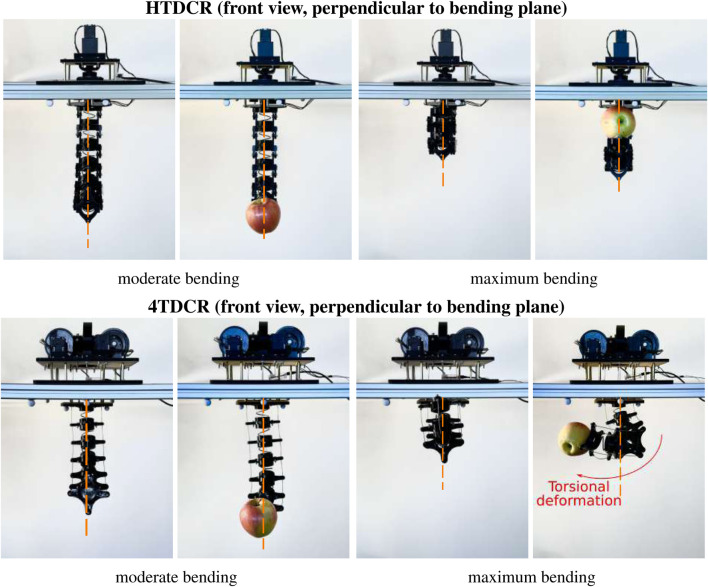
Illustrative example of torsional deformation Frontview comparison of the HTDCR and 4TDCR prototypes with and without a 186 g apple at the tip. Two bendings were considered (moderate and maximum).

Figure 6 shows an example of the behavior of the HTDCR vs. 4TDCR when a load is applied to the tip. We note that, unlike the HTDCR, the 4TDCR undergoes torsional deformation under load. To characterize the deformation, the shape of the body was measured at multiple points with reflective markers along the backbone, including the tip. Measurements were obtained with and without load for 18 random bendings, with 3 trials each, leading to a total of 108 measurements for each prototype. The experimental data was analyzed by comparing the measured positions with predictions from the constant curvature model and with measurements obtained with the 4TDCR.

**FIGURE 7:**
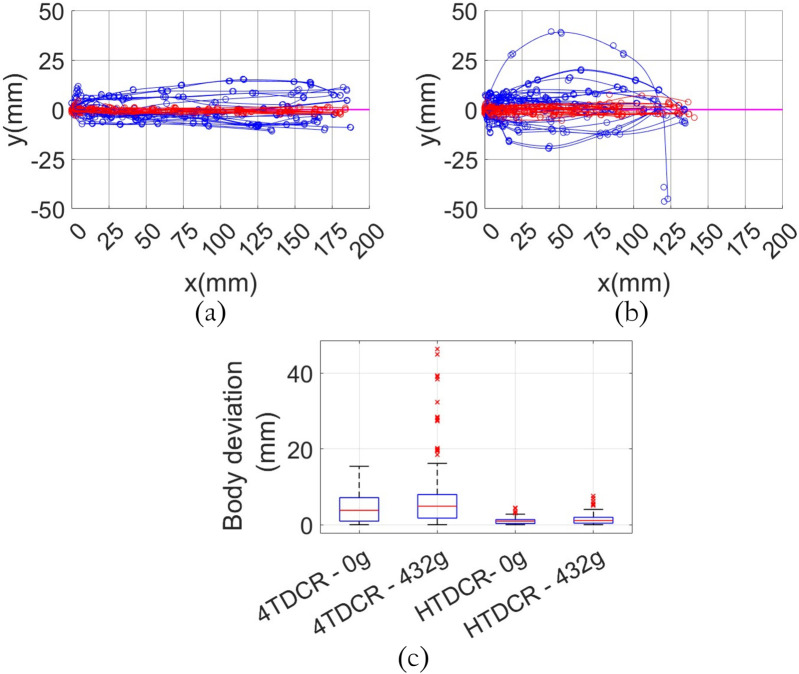
Body deviation out of the bending plane for 4TDCR (plots in blue) vs. HTDCR (plots in red) with and without tip load. The superimposed plots in panel a and b were obtained by rotating the measured positions so that the resulting bending plane corresponds to ϕ=0 . **(a)** Without tip load from the top view with maximum deviation of 15 mm for the 4TDCR and 4 mm for the HTDCR **(b)** With maximum load (432 g) from the top view with maximum deviation of 46 mm for the 4TDCR and 7 mm for the HTDCR. **(c)** Distribution of the body deviations for both prototypes with and without tip load.

Body deviation error is defined as the perpendicular distance between the center line of the backbone and the bending plane so that it is zero when the center line of the backbone belongs to the bending plane. In other words, it represents the displacement of the backbone out of the bending plane as a result of torsional deformation. Top view ( xy plane) of body deviation is displayed in Figure 7 for HTDCR (red data) and for 4TDCR (blue data). Without load (Figure 7A), maximum body deviation is 4 mm for HTDCR vs. 15 mm for 4TDCR. With load (Figure 7B), it is 7 mm for HTDCR vs. 46 mm for 4TDCR. Body deviation is much higher for 4TDCR than for HTDCR with or without load (P < 0.001, Kolmogorov-Smirnov test, Figure 7C). Although both prototypes exhibited the highest deviation in the fully bent position under maximum load, the body deviation for the HTDCR is negligeable (<8 mm) unlike the 4TDCR which presents significant torsional deformations (>4 cm).

### 3.4 Tip deflection analysis

**FIGURE 8:**
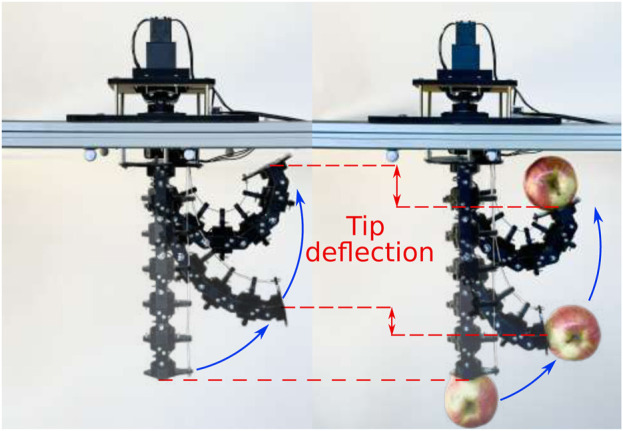
Illustrative example of tip deflection for HTDCR. Lateral view, facing the bending plane, for HTDCR with and without load.

**FIGURE 9:**
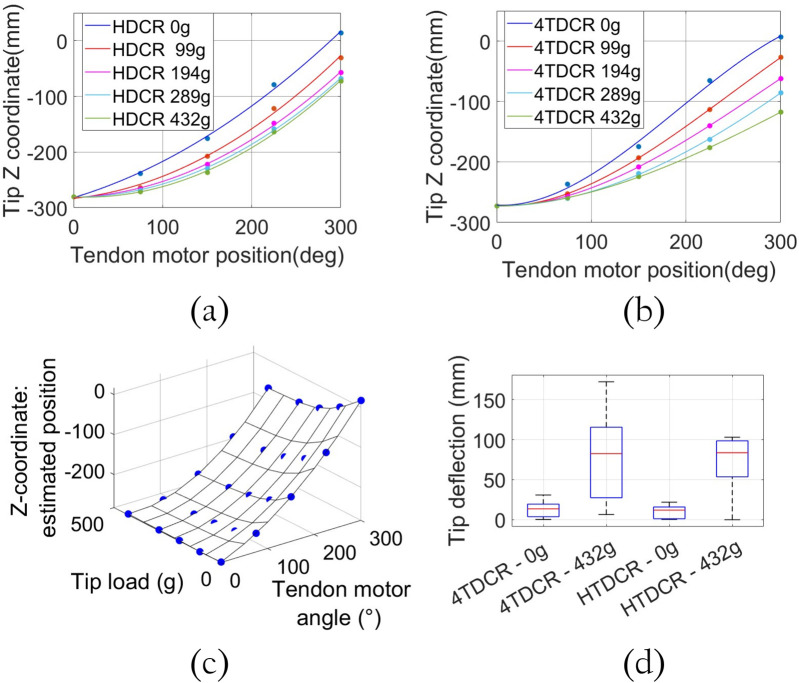
Tip deflection analysis with diffent loads and bending positions. Tip deflection for HTDCR **(a)** vs. 4TDCR **(b)** Tip deflection is measured from initial to maximum tendon motor position as the Z -coordinate of the tip when applying different loads. **(c)** HTDCR tip deflection vs. tip load and bending angle. A polynomial of degree 3 was fitted on the tendon mtoor angles and another of degree 4 on the tip loads. Altogether, it constitutes a 25 data (5 loads applied to the tip and 5 positions of the arm). Pearson correlation $R^{2}=0.9999$ , RMSE = 0.97 mm. **(d)** Distribution of the tip deflection in the bending plane of both prototypes with and without maximum tip load.

Tip deflection under load is simply defined as the difference in Z-coordinate obtained with and without load. Specifically, it refers to the change in the tip position within the bending plane due to the applied external load (see Figure 8). The first series of tests involved adding weight progressively to the tip, starting from no load, up to the maximum load of 432 g. The effects of the load was tested in different positions for the tendon servomotor starting from 0° to 300° in 75° increments. The motor position is directly correlated to the bending angle, with 0° corresponding to a fully extended position (no bending) and 300° to the maximum bending position. The same tests were conducted with the 4TDCR. As expected, tip deflection depends on the bending angle for both HTDCR and 4TDCR and increases as the arm bends further. Also, in Figure 9, it is observed that tip deflection increases with load due to the body flexibility retained within the bending plane of both prototypes. In the 4TDCR, the deflection increases proportionally with the load. In the HTDCR, the deflection rate is nonlinear and tip deflection stabilizes for higher loads. This is noticeable between the loads 194g, 289g and 432 g in Figure 9A. Actually, the tip deflection can be predicted accurately from the tip load and the tendon motor angle (Root Mean Squared Error (RMSE) in the mm range, Figure 9C).

### 3.5 Tip error analysis

In previous analysis, body deviation and tip deflection were measured with respect to the bending plane. Here we analyze how deviation and deflection errors translate into global tip position (XYZ) and tip orientation (ϕ) . The error percentage in tip position was calculated for both prototypes as in Equation 8:

$$\%Error=\left(\frac{Error_{\mathrm{calculated}}}{L}\right)*100$$ (8)

**FIGURE 10:**
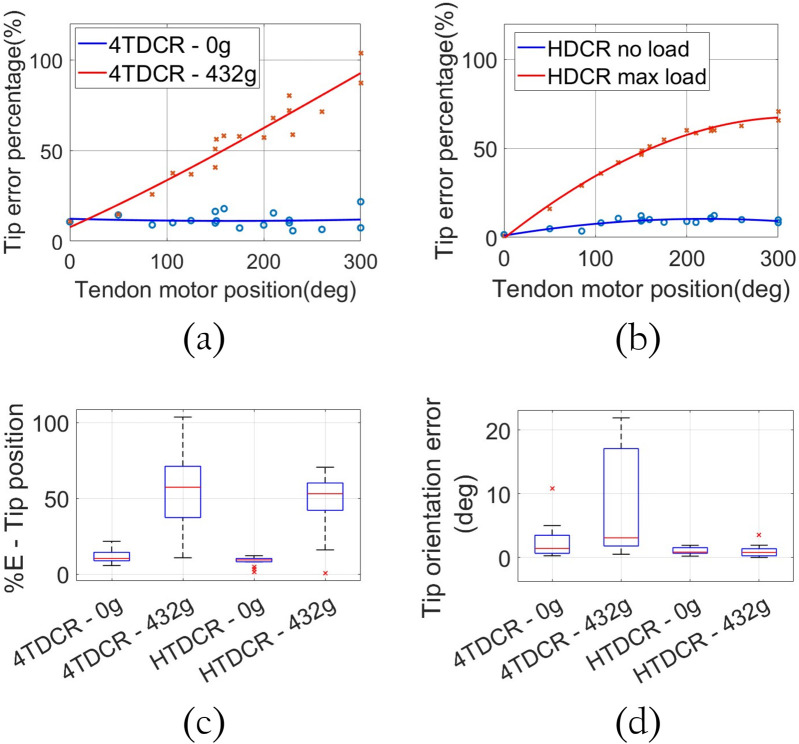
Comparison of the tip position (XYZ) and tip orientation (ϕ) error percentage for the 4TDCR and the HTDCR without load (blue data) and with maximum tip load (red data). **(a)** 4TDCR calculated tip position error percentage. **(b)** HTDCR calculated tip position error percentage. **(c)** Distribution of the tip position error percentage for both prototypes. **(d)** Distribution of the tip orientation (ϕ) error for both prototypes with and without maximum load (°) .

Where $Error_{\mathrm{calculated}}$ represents the error between the measured tip position and the one given by the model, and L is the length of the robot body (Rao et al., 2021; Amanov et al., 2021; He et al., 2013; Rucker and Webster, 2011). The error percentage was plotted for each prototype under with and without load and a polynomial fit was applied. Without external load (blue data in Figures 10A, B for 4TDCR and HTDCR, respectively), both prototypes exhibit similar error values. Under maximum load (red data in Figure 10A), the global tip error in the 4TDCR increases linearly with the bending angle (tendon motor position). In contrast, the error saturates in the HTDCR after the tendon motor reaches 200 ° (red data in Figure 10B). For example, the error at 300 ° for the HTDCR is 32% less from that of the 4TDCR. However, the most significant improvement is observed in the tip orientation (ϕ) in Figure 10D, which shows the distribution of orientation error at the tip. The 4TDCR reaches a maximum orientation error of 21.92 ° , while the HTDCR reduces this error significantly to just 3.6 ° . This reduction reflects the HTDCR’s enhanced stability and control under load, minimizing torsional deviation outside the bending plane.

## 4 Discussion

This study presents the design and validation of a Hybrid 1-Tendon Driven Continuum Robot (HTDCR), incorporating revolute joints to the structure for avoiding the effects of torsion under external loads, which remains a persistent challenge in continuum robotics. Two series of experiments were conducted on the HTDCR with varying loads. The data obtained were compared with those from a conventional 4-Tendon Driven Continuum Robot (4TDCR). The first test involved a progressive loading on the tip, while the second tested random configurations with and without maximum load.

The results of the experiments demonstrate that the mechanical constraint introduced by the revolute joints effectively reduces torsional effects, allowing for significantly improved control of the robot’s orientation when subjected to various loads. This design feature enhances the HTDCR’s stability and orientation control, especially under increasing load conditions (Figure 10D) having a maximum orientation error of 3.6°. While the integration of revolute joints significantly reduces torsional effects, deflection within the bending plane persists under external load due to the robot flexible backbone. This highlights an important issue to address for future research in order to explore ways to further balance flexibility with rigidity in continuum robots. One line of research would be to implement a tip deflection compensation system based on the established relationship between the tendon motor position and the applied load to obtain an estimation of the deflection, providing a better understanding of the position when the body deforms.

**FIGURE 11:**
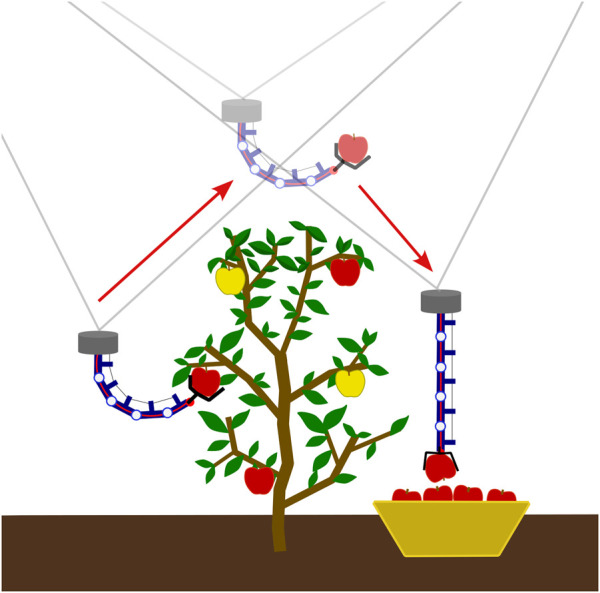
Harvesting scenario with the HTDCR used as the end-effector of a cable-driven parallel robot.

The HTDCR is particularly advantageous for applications requiring relatively high loads in continuum robotics, e.g., harvesting small-to medium-sized fruits and vegetables, such as apples with an average weight of 200g. Future work will concentrate on integrating the HTDCR with a cable-driven robot (Pott, 2018; Pannequin et al., 2020) or a quadrirotor for performing pest detection, crop surveillance, and harvesting as depicted in Figure 11.

## Data Availability

The raw data supporting the conclusions of this article will be made available by the authors, without undue reservation.
